# Methyl 3-(2-chlorophenyl)-2-(1*H*-indol-3-ylmethyl)-5-[1-(4-methoxyphenyl)-4-oxo-3-phenylazetidin-2-yl]-4-nitropyr­rolidine-2-carboxylate

**DOI:** 10.1107/S1600536808013585

**Published:** 2008-05-14

**Authors:** S. Nirmala, E. Theboral Sugi Kamala, L. Sudha, N. Arumugam, R. Raghunathan

**Affiliations:** a Department of Physics, Easwari Engineering College, Ramapuram, Chennai 600 089, India; b Department of Physics, SRM University, Ramapuram Campus, Chennai 600 089, India; c Department of Organic Chemistry, University of Madras, Guindy Campus, Chennai 600 025, India

## Abstract

In the mol­ecule of the title compound, C_37_H_33_ClN_4_O_6_, the four-membered *β*-lactam ring is essentially planar and is oriented at dihedral angles of 30.0 (1), 76.3 (1) and 30.9 (1)° with respect to the methoxy­phenyl ring, the phenyl ring and the indole unit, respectively. The pyrrolidine ring adopts a twist conformation. Intra­molecular C—H⋯Cl and C—H⋯O hydrogen bonds result in the formation of two five- and one six-membered rings. In the crystal structure, inter­molecular C—H⋯O and N—H⋯O hydrogen bonds link the mol­ecules. A weak π⋯π inter­action between the pyrrole rings further stabilizes the structure, with a centroid–centroid distance of 3.806 (2) Å.

## Related literature

For general background, see: Bruggink (2001 ▶); Morin & Gorman (1982 ▶); Katritzky *et al.* (1996 ▶); Georg (1993 ▶); Coyne *et al.* (2007 ▶); Dobrowolski *et al.* (2004 ▶); Cha *et al.* (2006 ▶). For related literature, see: Bhaskaran *et al.* (2006 ▶); Kamala *et al.* (2008 ▶); Ülkü *et al.* (1997 ▶). For ring puckering parameters, see: Cremer & Pople (1975 ▶). For asymmetry parameters, see: Nardelli (1995 ▶).
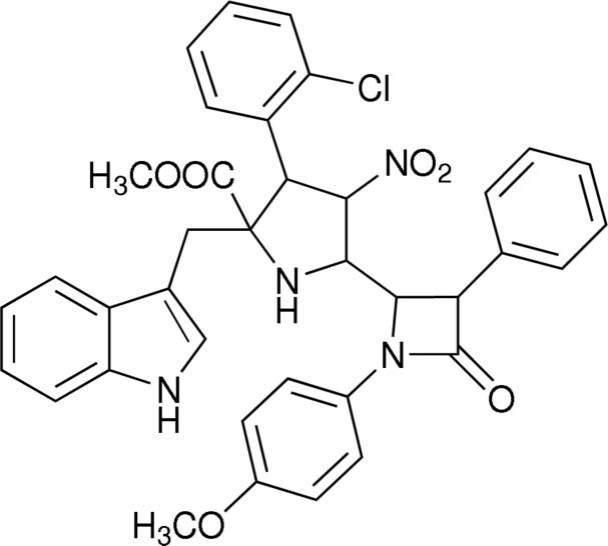


## Experimental

### 

#### Crystal data

C_37_H_33_ClN_4_O_6_
*M* *_r_* = 665.12
Triclinic, 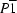

*a* = 10.399 (3) Å
*b* = 12.500 (3) Å
*c* = 14.211 (3) Å
α = 93.766 (6)°
β = 99.962 (6)°
γ = 114.066 (5)°
*V* = 1642.1 (7) Å^3^
*Z* = 2
Mo *K*α radiation
μ = 0.17 mm^−1^
*T* = 293 (2) K
0.30 × 0.20 × 0.16 mm

#### Data collection

Bruker Kappa APEX2 CCD diffractometer
Absorption correction: multi-scan (Blessing, 1995 ▶) *T*_min_ = 0.951, *T*_max_ = 0.973
25481 measured reflections
5563 independent reflections
3770 reflections with *I* > 2σ(*I*)
*R*_int_ = 0.057

#### Refinement

*R*[*F* ^2^ > 2σ(*F* ^2^)] = 0.067
*wR*(*F* ^2^) = 0.317
*S* = 1.10
5563 reflections
433 parameters
H-atom parameters constrained
Δρ_max_ = 0.51 e Å^−3^
Δρ_min_ = −0.64 e Å^−3^

### 

Data collection: *APEX2* (Bruker, 2004 ▶); cell refinement: *APEX2* and *SAINT* (Bruker, 2004 ▶); data reduction: *SAINT* and *XPREP* (Bruker, 2004 ▶); program(s) used to solve structure: *SIR92* (Altomare *et al.*, 1993 ▶); program(s) used to refine structure: *SHELXL97* (Sheldrick, 2008 ▶); molecular graphics: *ORTEP-3* (Farrugia, 1997 ▶); software used to prepare material for publication: *PLATON* (Spek, 2003 ▶).

## Supplementary Material

Crystal structure: contains datablocks I, global. DOI: 10.1107/S1600536808013585/hk2460sup1.cif

Structure factors: contains datablocks I. DOI: 10.1107/S1600536808013585/hk2460Isup2.hkl

Additional supplementary materials:  crystallographic information; 3D view; checkCIF report

## Figures and Tables

**Table 1:** Hydrogen-bond geometry (Å, °)

*D*—H⋯*A*	*D*—H	H⋯*A*	*D*⋯*A*	*D*—H⋯*A*
C11—H11⋯Cl1	0.98	2.57	3.095 (4)	114
C11—H11⋯O3	0.98	2.37	2.786 (4)	105
C22—H22⋯O5	0.93	2.59	3.080 (6)	113
C14—H14⋯O4^i^	0.98	2.53	3.443 (5)	154
C34—H34⋯O4^ii^	0.93	2.59	3.414 (6)	148
N1—H1*A*⋯O6^iii^	0.86	2.14	2.982 (5)	167

Symmetry codes: (i) 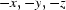; (ii) 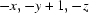; (iii) 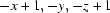.

**Fig. 1.**
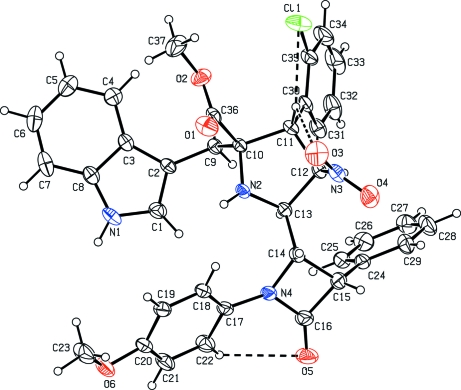
The molecular structure of the title molecule, with the atom-numbering scheme. Displacement ellipsoids are drawn at the 30% probability level. Hydrogen bonds are shown as dashed lines.

**Fig. 2.**
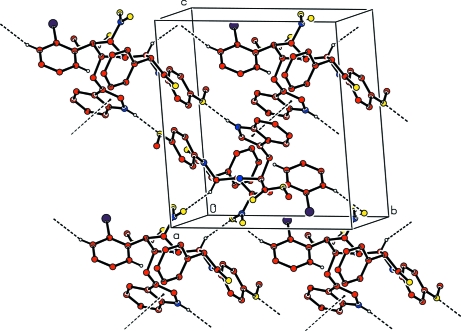
A partial packing diagram for (I). Hydrogen bonds are shown as dashed lines. H atoms not involed in hydrogen bondings have been omitted for clarity.

## supplementary crystallographic information

### Comment

*β*-Lactams are one of the best known and most extensively studied class of compounds due to their biological activity (Bruggink, 2001; Morin & Gorman, 1982; Katritzky *et al.*, 1996; Georg, 1993). The *β*-lactam class of drugs have revolutionized treatment in medicine (Coyne *et al.*, 2007). In the late 1970's and early 1980's, the first class of the monocyclic *β*-lactam antibacterial agents were found in natural sources (Dobrowolski *et al.*, 2004). All *β*-lactams are based on a *β*-lactam ring responsible for the antibacterial activity and variable side chains that account for the major differences in their chemical and pharmocological properties (Cha *et al.*, 2006). We report herein the crystal structure of the title compound, (I).

In the title compound, (I), (Fig. 1) the four-membered *β*-lactam ring A (N4/C14-C16) is nearly planar, with a maximum deviation of 0.038 (4) Å for atom N1. The C14-C15 [1.581 (4) Å] and C15-C16 [1.523 (5) Å] bonds agree with those observed in similar structures (Bhaskaran *et al.*, 2006; Kamala *et al.*, 2008). The C14-C15-C16 [84.6 (2)°] bond angle is comparable to the corresponding value [87.0 (3)°)] in a related structure (Ülkü *et al.*, 1997). The sum of the bond angles around atom N4 [355.6 (3)°] indicates *sp*^2^ hybridization. The planar rings A, B (C17-C22) and C (C24-C29) are oriented at dihedral angles of A/B = 30.0 (1)°, A/C = 76.3 (1)° and B/C = 50.2 (1)°. The planar indole moiety is oriented with respect to rings A, C and D (C30-C35) at dihedral angles of 30.9 (1)°, 73.0 (1)° and 70.7 (1)°, respectively. The pyrrolidine ring E (N2/C10-C13) adopts a twisted conformation, with asymmetry [Δ*C*_2_ (C11) = 0.011 (1), Δ*C*_s_ (C13) = 0.085 (2)] (Nardelli, 1995) and puckering [*q*_2_ = 0.402 (3) Å and *φ*= -21.1 (4)°] (Cremer & Pople, 1975) parameters. Atom N2 deviates from the mean plane of (N2/C10-C12) by 0.553 (7) Å.

The intramolecular C-H···Cl and C-H···O hydrogen bonds (Table 1) result in the formation of two five- and one six-membered rings: F (O3/N3/C11/H11A/C12), G (Cl1/C11/H11/C30/C39) and H (O5/N4/C16/C17/C22/H22), respectively. In the crystal structure, intermolecular C-H···O and N-H···O hydrogen bonds (Table 1) link the molecules (Fig. 2), in which they may be effective in the stabilization of the structure. A weak π—π interaction between (N1/C1-C3/C8) rings at x, y, z and 1 - x, 1 - y, 1 - z further stabilize the structure, with a centroid-centroid distance of 3.806 (2) Å.

### Experimental

For the preparation of the title compound, *β*-Lactam aldehyde (1.0 mol) was treated with tryptophan methylester hydrochloride (1.0 mol) in the presence of Et_3_N (2.5 mol) and anhydrous MgSO_4_ (2.0 g) in dry dichloromethane (10 ml) at room temperature for 12 h to give the imine. The imine was washed with water and dried over Na_2_SO_4_. The solvent was evaporated under vacuum. The imine (1.0 mol) was then strirred with silver (I) acetate and *p*-chloro nitrostyrene (1.0 mol) in the presence of Et_3_N (1.2 mol) and molecular sieves in dry toluene (30 ml) at room temperature for 12 h. The reaction mixture was filtered through a plug celite. The solvent was evaporated under reduced pressure and the residue was subjected to column chromatography on silica gel (100-200 mesh), with hexane-ethylacetate (7:3) as eluent to give the product. The compound was recrystallized from ethylacetate.

### Refinement

H atoms were positioned geometrically, with N-H = 0.86 Å (for NH) and C-H = 0.93, 0.98, 0.97 and 0.96 Å for aromatic, methine, methylene and methyl H, respectively, and constrained to ride on their parent atoms with U_iso_(H) = xU_eq_(C,N), where x = 1.5 for methyl H, and x = 1.2 for all other H atoms.

### Crystal data

**Table:**

C_37_H_33_ClN_4_O_6_	*Z* = 2
*M**_r_* = 665.12	*F*_000_ = 696
Triclinic, *P*1	*D*_x_ = 1.345 Mg m^−^^3^
Hall symbol: -P 1	Mo *K*α radiation λ = 0.71073 Å
*a* = 10.399 (3) Å	Cell parameters from 8315 reflections
*b* = 12.500 (3) Å	θ = 2.5–31.6º
*c* = 14.211 (3) Å	µ = 0.17 mm^−^^1^
α = 93.766 (6)º	*T* = 293 (2) K
β = 99.962 (6)º	Prism, colourless
γ = 114.066 (5)º	0.30 × 0.20 × 0.16 mm
*V* = 1642.1 (7) Å^3^	

### Data collection

**Table:**

Bruker KAPPA APEX2 CCD diffractometer	5563 independent reflections
Radiation source: fine-focus sealed tube	3770 reflections with *I* > 2σ(*I*)
Monochromator: graphite	*R*_int_ = 0.057
*T* = 294(2) K	θ_max_ = 25.0º
ω and φ scans	θ_min_ = 1.5º
Absorption correction: multi-scan(Blessing, 1995)	*h* = −12→12
*T*_min_ = 0.951, *T*_max_ = 0.973	*k* = −14→14
25481 measured reflections	*l* = −16→16

### Refinement

**Table:**

Refinement on *F*^2^	Secondary atom site location: difference Fourier map
Least-squares matrix: full	Hydrogen site location: inferred from neighbouring sites
*R*[*F*^2^ > 2σ(*F*^2^)] = 0.067	H-atom parameters constrained
*wR*(*F*^2^) = 0.317	*w* = 1/[σ^2^(*F*_o_^2^) + (0.2*P*)^2^] where *P* = (*F*_o_^2^ + 2*F*_c_^2^)/3
*S* = 1.10	(Δ/σ)_max_ < 0.001
5563 reflections	Δρ_max_ = 0.51 e Å^−^^3^
433 parameters	Δρ_min_ = −0.64 e Å^−^^3^
Primary atom site location: structure-invariant direct methods	Extinction correction: none

### Special details

**Table:**

Geometry. All e.s.d.'s (except the e.s.d. in the dihedral angle between two l.s. planes) are estimated using the full covariance matrix. The cell e.s.d.'s are taken into account individually in the estimation of e.s.d.'s in distances, angles and torsion angles; correlations between e.s.d.'s in cell parameters are only used when they are defined by crystal symmetry. An approximate (isotropic) treatment of cell e.s.d.'s is used for estimating e.s.d.'s involving l.s. planes.
Refinement. Refinement of F^2^ against ALL reflections. The weighted R-factor wR and goodness of fit S are based on F^2^, conventional R-factors R are based on F, with F set to zero for negative F^2^. The threshold expression of F^2^ > 2sigma(F^2^) is used only for calculating R-factors(gt) etc. and is not relevant to the choice of reflections for refinement. R-factors based on F^2^ are statistically about twice as large as those based on F, and R- factors based on ALL data will be even larger.

### Fractional atomic coordinates and isotropic or equivalent isotropic displacement parameters (Å^2^)

**Table:**

	*x*	*y*	*z*	*U*_iso_*/*U*_eq_	
Cl1	0.27147 (16)	0.62053 (13)	0.03791 (11)	0.0912 (5)	
O1	0.4619 (3)	0.3522 (3)	0.1048 (2)	0.0620 (8)	
O2	0.4973 (3)	0.5325 (3)	0.16978 (19)	0.0549 (7)	
O3	0.1219 (3)	0.2147 (3)	−0.0280 (2)	0.0690 (9)	
O4	−0.0986 (3)	0.1137 (3)	−0.02304 (19)	0.0646 (8)	
O5	−0.1691 (3)	−0.1217 (3)	0.3043 (3)	0.0861 (12)	
O6	0.4562 (3)	−0.1339 (3)	0.4179 (2)	0.0647 (9)	
N1	0.5503 (4)	0.3211 (3)	0.4608 (2)	0.0500 (8)	
H1A	0.5602	0.2674	0.4921	0.060*	
N2	0.2433 (3)	0.2437 (2)	0.19400 (18)	0.0340 (6)	
H2	0.2943	0.2039	0.1976	0.041*	
N3	0.0198 (3)	0.1926 (3)	0.0102 (2)	0.0441 (8)	
N4	0.0375 (3)	0.0089 (2)	0.2561 (2)	0.0406 (7)	
C1	0.4247 (4)	0.3105 (3)	0.4029 (2)	0.0452 (9)	
H1	0.3372	0.2435	0.3914	0.054*	
C2	0.4455 (3)	0.4115 (3)	0.3646 (2)	0.0344 (7)	
C3	0.5947 (3)	0.4900 (3)	0.4004 (2)	0.0355 (8)	
C4	0.6834 (4)	0.6067 (3)	0.3897 (2)	0.0464 (9)	
H4	0.6465	0.6488	0.3504	0.056*	
C5	0.8251 (4)	0.6574 (4)	0.4383 (3)	0.0614 (11)	
H5	0.8838	0.7347	0.4320	0.074*	
C6	0.8836 (4)	0.5963 (5)	0.4969 (3)	0.0669 (13)	
H6	0.9802	0.6335	0.5289	0.080*	
C7	0.8008 (4)	0.4815 (5)	0.5083 (3)	0.0592 (12)	
H7	0.8402	0.4400	0.5465	0.071*	
C8	0.6570 (4)	0.4301 (3)	0.4609 (2)	0.0411 (8)	
C9	0.3323 (3)	0.4348 (3)	0.3002 (2)	0.0340 (7)	
H9A	0.3665	0.5194	0.2999	0.041*	
H9B	0.2457	0.4079	0.3260	0.041*	
C10	0.2943 (3)	0.3712 (3)	0.1948 (2)	0.0310 (7)	
C11	0.1669 (3)	0.3868 (3)	0.1245 (2)	0.0335 (7)	
H11	0.1957	0.4033	0.0630	0.040*	
C12	0.0405 (3)	0.2628 (3)	0.1066 (2)	0.0331 (7)	
H12	−0.0490	0.2678	0.1139	0.040*	
C13	0.0886 (3)	0.1980 (3)	0.1857 (2)	0.0315 (7)	
H13	0.0732	0.2268	0.2468	0.038*	
C14	0.0125 (3)	0.0649 (3)	0.1707 (2)	0.0357 (7)	
H14	0.0309	0.0299	0.1137	0.043*	
C15	−0.1531 (3)	0.0061 (3)	0.1733 (3)	0.0458 (9)	
H15	−0.2101	−0.0532	0.1159	0.055*	
C16	−0.1057 (4)	−0.0505 (3)	0.2560 (3)	0.0519 (10)	
C17	0.1534 (3)	−0.0169 (3)	0.2985 (2)	0.0378 (8)	
C18	0.2642 (3)	−0.0060 (3)	0.2526 (2)	0.0393 (8)	
H18	0.2685	0.0256	0.1952	0.047*	
C19	0.3685 (3)	−0.0420 (3)	0.2917 (3)	0.0430 (8)	
H19	0.4434	−0.0337	0.2610	0.052*	
C20	0.3619 (4)	−0.0899 (3)	0.3756 (3)	0.0460 (9)	
C21	0.2550 (4)	−0.0962 (4)	0.4239 (3)	0.0544 (10)	
H21	0.2533	−0.1250	0.4826	0.065*	
C22	0.1513 (4)	−0.0600 (4)	0.3857 (3)	0.0509 (10)	
H22	0.0796	−0.0645	0.4185	0.061*	
C23	0.5611 (5)	−0.1357 (5)	0.3669 (4)	0.0807 (16)	
H23A	0.6198	−0.1683	0.4029	0.121*	
H23B	0.6208	−0.0564	0.3585	0.121*	
H23C	0.5137	−0.1838	0.3048	0.121*	
C24	−0.2264 (3)	0.0835 (3)	0.1965 (3)	0.0418 (9)	
C25	−0.1948 (4)	0.1455 (3)	0.2876 (3)	0.0461 (9)	
H25	−0.1276	0.1389	0.3365	0.055*	
C26	−0.2613 (4)	0.2174 (4)	0.3075 (3)	0.0555 (10)	
H26	−0.2388	0.2582	0.3696	0.067*	
C27	−0.3605 (4)	0.2289 (4)	0.2362 (3)	0.0605 (11)	
H27	−0.4052	0.2774	0.2495	0.073*	
C28	−0.3929 (4)	0.1674 (4)	0.1442 (3)	0.0618 (12)	
H28	−0.4591	0.1752	0.0953	0.074*	
C29	−0.3278 (4)	0.0951 (4)	0.1250 (3)	0.0537 (10)	
H29	−0.3517	0.0531	0.0632	0.064*	
C30	0.1230 (3)	0.4815 (3)	0.1585 (2)	0.0386 (8)	
C31	0.0321 (4)	0.4623 (4)	0.2234 (3)	0.0473 (9)	
H31	0.0003	0.3906	0.2472	0.057*	
C32	−0.0116 (5)	0.5464 (4)	0.2530 (3)	0.0664 (13)	
H32	−0.0731	0.5307	0.2957	0.080*	
C33	0.0349 (7)	0.6529 (5)	0.2198 (4)	0.0842 (18)	
H33	0.0062	0.7101	0.2406	0.101*	
C34	0.1243 (6)	0.6751 (4)	0.1557 (4)	0.0775 (16)	
H34	0.1564	0.7477	0.1333	0.093*	
C35	0.1674 (4)	0.5889 (3)	0.1239 (3)	0.0537 (10)	
C36	0.4283 (3)	0.4159 (3)	0.1513 (2)	0.0384 (8)	
C37	0.6263 (4)	0.5862 (5)	0.1315 (3)	0.0768 (15)	
H37A	0.6678	0.6708	0.1491	0.115*	
H37B	0.6014	0.5664	0.0623	0.115*	
H37C	0.6950	0.5570	0.1577	0.115*	

### Atomic displacement parameters (Å^2^)

**Table:**

	*U*^11^	*U*^22^	*U*^33^	*U*^12^	*U*^13^	*U*^23^
Cl1	0.1027 (10)	0.0749 (10)	0.1001 (11)	0.0317 (8)	0.0319 (8)	0.0577 (8)
O1	0.0716 (17)	0.076 (2)	0.0706 (19)	0.0510 (17)	0.0408 (14)	0.0291 (16)
O2	0.0497 (14)	0.0547 (19)	0.0521 (16)	0.0091 (13)	0.0214 (11)	0.0149 (13)
O3	0.081 (2)	0.083 (2)	0.0504 (17)	0.0411 (18)	0.0232 (15)	−0.0032 (16)
O4	0.0702 (18)	0.060 (2)	0.0491 (16)	0.0255 (16)	−0.0112 (13)	−0.0109 (14)
O5	0.0532 (16)	0.084 (2)	0.149 (3)	0.0374 (16)	0.0485 (19)	0.078 (2)
O6	0.0597 (15)	0.083 (2)	0.079 (2)	0.0484 (16)	0.0245 (14)	0.0487 (17)
N1	0.0702 (19)	0.057 (2)	0.0369 (16)	0.0401 (18)	0.0100 (14)	0.0201 (15)
N2	0.0349 (12)	0.0346 (16)	0.0398 (15)	0.0216 (12)	0.0076 (10)	0.0116 (12)
N3	0.0589 (18)	0.0459 (19)	0.0339 (16)	0.0323 (16)	0.0013 (13)	0.0045 (14)
N4	0.0355 (13)	0.0350 (17)	0.0586 (18)	0.0193 (12)	0.0137 (12)	0.0177 (14)
C1	0.0529 (18)	0.047 (2)	0.0362 (18)	0.0206 (17)	0.0092 (14)	0.0129 (17)
C2	0.0423 (16)	0.040 (2)	0.0277 (16)	0.0230 (15)	0.0104 (12)	0.0111 (14)
C3	0.0443 (16)	0.044 (2)	0.0240 (15)	0.0256 (15)	0.0068 (12)	0.0030 (14)
C4	0.0500 (18)	0.048 (2)	0.0393 (19)	0.0186 (17)	0.0110 (15)	0.0044 (17)
C5	0.049 (2)	0.058 (3)	0.061 (3)	0.0101 (19)	0.0095 (17)	−0.003 (2)
C6	0.0400 (18)	0.100 (4)	0.050 (2)	0.025 (2)	−0.0003 (16)	−0.003 (2)
C7	0.058 (2)	0.098 (4)	0.037 (2)	0.053 (3)	0.0016 (16)	0.006 (2)
C8	0.0518 (18)	0.050 (2)	0.0278 (16)	0.0287 (18)	0.0060 (13)	0.0033 (15)
C9	0.0360 (14)	0.0389 (19)	0.0332 (17)	0.0213 (14)	0.0092 (12)	0.0071 (14)
C10	0.0329 (14)	0.0348 (19)	0.0317 (16)	0.0195 (13)	0.0082 (11)	0.0103 (14)
C11	0.0425 (15)	0.0373 (19)	0.0281 (16)	0.0229 (14)	0.0091 (12)	0.0115 (14)
C12	0.0384 (15)	0.0376 (19)	0.0307 (16)	0.0239 (14)	0.0069 (12)	0.0046 (14)
C13	0.0357 (14)	0.0324 (18)	0.0315 (16)	0.0196 (13)	0.0070 (11)	0.0055 (13)
C14	0.0412 (15)	0.0311 (18)	0.0406 (18)	0.0221 (14)	0.0070 (12)	0.0050 (14)
C15	0.0375 (16)	0.039 (2)	0.064 (2)	0.0211 (15)	0.0078 (14)	0.0087 (17)
C16	0.0438 (18)	0.042 (2)	0.080 (3)	0.0235 (17)	0.0197 (18)	0.025 (2)
C17	0.0420 (16)	0.0296 (18)	0.0468 (19)	0.0188 (14)	0.0109 (14)	0.0120 (15)
C18	0.0402 (15)	0.037 (2)	0.0451 (19)	0.0187 (15)	0.0109 (13)	0.0159 (16)
C19	0.0431 (17)	0.043 (2)	0.051 (2)	0.0214 (16)	0.0180 (14)	0.0179 (17)
C20	0.0457 (17)	0.044 (2)	0.054 (2)	0.0232 (16)	0.0111 (15)	0.0234 (18)
C21	0.060 (2)	0.070 (3)	0.049 (2)	0.037 (2)	0.0207 (17)	0.033 (2)
C22	0.0537 (19)	0.061 (3)	0.052 (2)	0.0312 (19)	0.0237 (16)	0.0249 (19)
C23	0.070 (3)	0.103 (4)	0.116 (4)	0.066 (3)	0.043 (3)	0.063 (3)
C24	0.0356 (15)	0.038 (2)	0.057 (2)	0.0193 (14)	0.0119 (14)	0.0158 (17)
C25	0.0439 (17)	0.047 (2)	0.050 (2)	0.0210 (16)	0.0104 (15)	0.0155 (18)
C26	0.057 (2)	0.062 (3)	0.060 (2)	0.032 (2)	0.0248 (18)	0.017 (2)
C27	0.059 (2)	0.064 (3)	0.085 (3)	0.044 (2)	0.032 (2)	0.027 (2)
C28	0.057 (2)	0.080 (3)	0.071 (3)	0.047 (2)	0.0166 (19)	0.031 (2)
C29	0.0485 (18)	0.064 (3)	0.056 (2)	0.0327 (19)	0.0065 (16)	0.013 (2)
C30	0.0435 (16)	0.041 (2)	0.0355 (17)	0.0263 (15)	−0.0031 (13)	0.0077 (15)
C31	0.057 (2)	0.052 (2)	0.045 (2)	0.0381 (19)	0.0046 (15)	0.0036 (17)
C32	0.074 (3)	0.080 (3)	0.059 (3)	0.056 (3)	−0.004 (2)	−0.008 (2)
C33	0.106 (4)	0.064 (3)	0.090 (4)	0.067 (3)	−0.025 (3)	−0.019 (3)
C34	0.093 (3)	0.041 (3)	0.093 (4)	0.039 (3)	−0.020 (3)	0.007 (2)
C35	0.057 (2)	0.037 (2)	0.062 (2)	0.0219 (17)	−0.0066 (17)	0.0126 (18)
C36	0.0404 (16)	0.053 (2)	0.0320 (17)	0.0278 (17)	0.0102 (13)	0.0177 (16)
C37	0.052 (2)	0.097 (4)	0.061 (3)	0.004 (2)	0.024 (2)	0.027 (3)

### Geometric parameters (Å, °)

**Table:**

N1—H1A	0.8600	C17—C18	1.384 (5)
N2—H2	0.8600	C17—N4	1.418 (4)
N3—O4	1.208 (4)	C18—C19	1.382 (4)
N3—O3	1.215 (4)	C18—H18	0.9300
C1—C2	1.358 (5)	C19—C20	1.368 (5)
C1—N1	1.369 (4)	C19—H19	0.9300
C1—H1	0.9300	C20—O6	1.380 (4)
C2—C3	1.431 (5)	C20—C21	1.383 (5)
C2—C9	1.501 (4)	C21—C22	1.375 (5)
C3—C4	1.408 (5)	C21—H21	0.9300
C3—C8	1.417 (4)	C22—H22	0.9300
C4—C5	1.371 (5)	C23—O6	1.417 (5)
C4—H4	0.9300	C23—H23A	0.9600
C5—C6	1.390 (7)	C23—H23B	0.9600
C5—H5	0.9300	C23—H23C	0.9600
C6—C7	1.377 (7)	C24—C25	1.378 (5)
C6—H6	0.9300	C24—C29	1.392 (4)
C7—C8	1.383 (5)	C25—C26	1.380 (5)
C7—H7	0.9300	C25—H25	0.9300
C8—N1	1.362 (5)	C26—C27	1.376 (5)
C9—C10	1.553 (4)	C26—H26	0.9300
C9—H9A	0.9700	C27—C28	1.384 (6)
C9—H9B	0.9700	C27—H27	0.9300
C10—N2	1.459 (4)	C28—C29	1.373 (5)
C10—C36	1.531 (4)	C28—H28	0.9300
C10—C11	1.603 (3)	C29—H29	0.9300
C11—C30	1.511 (4)	C30—C35	1.385 (5)
C11—C12	1.538 (5)	C30—C31	1.395 (5)
C11—H11	0.9800	C31—C32	1.374 (5)
C12—N3	1.511 (4)	C31—H31	0.9300
C12—C13	1.558 (4)	C32—C33	1.365 (8)
C12—H12	0.9800	C32—H32	0.9300
C13—N2	1.451 (4)	C33—C34	1.374 (8)
C13—C14	1.503 (4)	C33—H33	0.9300
C13—H13	0.9800	C34—C35	1.402 (6)
C14—N4	1.478 (4)	C34—H34	0.9300
C14—C15	1.581 (4)	C35—Cl1	1.730 (5)
C14—H14	0.9800	C36—O1	1.197 (4)
C15—C24	1.507 (4)	C36—O2	1.319 (4)
C15—C16	1.523 (5)	C37—O2	1.454 (4)
C15—H15	0.9800	C37—H37A	0.9600
C16—O5	1.206 (5)	C37—H37B	0.9600
C16—N4	1.365 (4)	C37—H37C	0.9600
C17—C22	1.383 (5)		
			
C36—O2—C37	116.4 (3)	C16—C15—C14	84.6 (2)
C20—O6—C23	116.9 (3)	C24—C15—H15	111.2
C8—N1—C1	109.2 (3)	C16—C15—H15	111.2
C8—N1—H1A	125.4	C14—C15—H15	111.2
C1—N1—H1A	125.4	O5—C16—N4	132.5 (3)
C13—N2—C10	105.6 (2)	O5—C16—C15	133.9 (3)
C13—N2—H2	127.2	N4—C16—C15	93.6 (3)
C10—N2—H2	127.2	C22—C17—C18	119.3 (3)
O4—N3—O3	123.6 (3)	C22—C17—N4	118.8 (3)
O4—N3—C12	116.9 (3)	C18—C17—N4	121.8 (3)
O3—N3—C12	119.5 (3)	C19—C18—C17	120.2 (3)
C16—N4—C17	128.4 (3)	C19—C18—H18	119.9
C16—N4—C14	94.5 (3)	C17—C18—H18	119.9
C17—N4—C14	132.7 (3)	C20—C19—C18	120.1 (3)
C2—C1—N1	110.6 (3)	C20—C19—H19	119.9
C2—C1—H1	124.7	C18—C19—H19	119.9
N1—C1—H1	124.7	C19—C20—O6	124.7 (3)
C1—C2—C3	106.0 (3)	C19—C20—C21	119.7 (3)
C1—C2—C9	126.1 (3)	O6—C20—C21	115.6 (3)
C3—C2—C9	127.9 (3)	C22—C21—C20	120.4 (3)
C4—C3—C8	118.1 (3)	C22—C21—H21	119.8
C4—C3—C2	134.6 (3)	C20—C21—H21	119.8
C8—C3—C2	107.3 (3)	C21—C22—C17	120.0 (3)
C5—C4—C3	118.9 (4)	C21—C22—H22	120.0
C5—C4—H4	120.6	C17—C22—H22	120.0
C3—C4—H4	120.6	O6—C23—H23A	109.5
C4—C5—C6	121.8 (4)	O6—C23—H23B	109.5
C4—C5—H5	119.1	H23A—C23—H23B	109.5
C6—C5—H5	119.1	O6—C23—H23C	109.5
C7—C6—C5	121.1 (4)	H23A—C23—H23C	109.5
C7—C6—H6	119.5	H23B—C23—H23C	109.5
C5—C6—H6	119.5	C25—C24—C29	118.1 (3)
C6—C7—C8	117.7 (3)	C25—C24—C15	121.7 (3)
C6—C7—H7	121.2	C29—C24—C15	120.3 (3)
C8—C7—H7	121.2	C24—C25—C26	121.1 (3)
N1—C8—C7	130.5 (3)	C24—C25—H25	119.4
N1—C8—C3	107.0 (3)	C26—C25—H25	119.4
C7—C8—C3	122.5 (4)	C27—C26—C25	120.4 (4)
C2—C9—C10	112.3 (2)	C27—C26—H26	119.8
C2—C9—H9A	109.1	C25—C26—H26	119.8
C10—C9—H9A	109.1	C26—C27—C28	119.1 (3)
C2—C9—H9B	109.1	C26—C27—H27	120.4
C10—C9—H9B	109.1	C28—C27—H27	120.4
H9A—C9—H9B	107.9	C29—C28—C27	120.3 (3)
N2—C10—C36	108.6 (2)	C29—C28—H28	119.8
N2—C10—C9	109.8 (2)	C27—C28—H28	119.8
C36—C10—C9	110.0 (2)	C28—C29—C24	121.0 (4)
N2—C10—C11	105.2 (2)	C28—C29—H29	119.5
C36—C10—C11	108.7 (2)	C24—C29—H29	119.5
C9—C10—C11	114.2 (2)	C35—C30—C31	117.5 (3)
C30—C11—C12	111.5 (2)	C35—C30—C11	121.3 (3)
C30—C11—C10	117.8 (2)	C31—C30—C11	121.2 (3)
C12—C11—C10	103.5 (2)	C32—C31—C30	121.9 (4)
C30—C11—H11	107.8	C32—C31—H31	119.1
C12—C11—H11	107.8	C30—C31—H31	119.1
C10—C11—H11	107.8	C33—C32—C31	120.2 (5)
N3—C12—C11	113.1 (3)	C33—C32—H32	119.9
N3—C12—C13	106.7 (2)	C31—C32—H32	119.9
C11—C12—C13	103.8 (2)	C32—C33—C34	119.7 (4)
N3—C12—H12	111.0	C32—C33—H33	120.2
C11—C12—H12	111.0	C34—C33—H33	120.2
C13—C12—H12	111.0	C33—C34—C35	120.4 (4)
N2—C13—C14	113.7 (2)	C33—C34—H34	119.8
N2—C13—C12	103.5 (2)	C35—C34—H34	119.8
C14—C13—C12	117.3 (2)	C30—C35—C34	120.3 (4)
N2—C13—H13	107.3	C30—C35—Cl1	122.1 (3)
C14—C13—H13	107.3	C34—C35—Cl1	117.5 (4)
C12—C13—H13	107.3	O1—C36—O2	125.4 (3)
N4—C14—C13	115.3 (3)	O1—C36—C10	123.6 (3)
N4—C14—C15	87.1 (2)	O2—C36—C10	111.0 (3)
C13—C14—C15	117.8 (2)	O2—C37—H37A	109.5
N4—C14—H14	111.5	O2—C37—H37B	109.5
C13—C14—H14	111.5	H37A—C37—H37B	109.5
C15—C14—H14	111.5	O2—C37—H37C	109.5
C24—C15—C16	116.4 (3)	H37A—C37—H37C	109.5
C24—C15—C14	119.7 (3)	H37B—C37—H37C	109.5
			
N1—C1—C2—C3	0.6 (4)	C16—C15—C24—C29	−150.9 (3)
N1—C1—C2—C9	−178.0 (3)	C14—C15—C24—C29	109.9 (4)
C1—C2—C3—C4	−178.8 (3)	C29—C24—C25—C26	−0.2 (5)
C9—C2—C3—C4	−0.3 (6)	C15—C24—C25—C26	179.1 (3)
C1—C2—C3—C8	−0.5 (4)	C24—C25—C26—C27	−0.3 (6)
C9—C2—C3—C8	178.1 (3)	C25—C26—C27—C28	0.1 (6)
C8—C3—C4—C5	−0.3 (5)	C26—C27—C28—C29	0.6 (6)
C2—C3—C4—C5	177.9 (4)	C27—C28—C29—C24	−1.1 (6)
C3—C4—C5—C6	0.6 (6)	C25—C24—C29—C28	0.9 (6)
C4—C5—C6—C7	0.1 (7)	C15—C24—C29—C28	−178.4 (4)
C5—C6—C7—C8	−1.2 (6)	C12—C11—C30—C35	−138.3 (3)
C6—C7—C8—N1	−178.1 (4)	C10—C11—C30—C35	102.2 (3)
C6—C7—C8—C3	1.5 (5)	C12—C11—C30—C31	39.4 (4)
C4—C3—C8—N1	178.9 (3)	C10—C11—C30—C31	−80.1 (4)
C2—C3—C8—N1	0.2 (3)	C35—C30—C31—C32	−0.5 (5)
C4—C3—C8—C7	−0.7 (5)	C11—C30—C31—C32	−178.3 (3)
C2—C3—C8—C7	−179.4 (3)	C30—C31—C32—C33	−0.7 (6)
C1—C2—C9—C10	−76.9 (4)	C31—C32—C33—C34	0.8 (7)
C3—C2—C9—C10	104.8 (4)	C32—C33—C34—C35	0.3 (7)
C2—C9—C10—N2	58.5 (3)	C31—C30—C35—C34	1.6 (5)
C2—C9—C10—C36	−61.0 (3)	C11—C30—C35—C34	179.4 (3)
C2—C9—C10—C11	176.5 (2)	C31—C30—C35—Cl1	−176.2 (2)
N2—C10—C11—C30	134.8 (3)	C11—C30—C35—Cl1	1.6 (5)
C36—C10—C11—C30	−109.0 (3)	C33—C34—C35—C30	−1.5 (6)
C9—C10—C11—C30	14.2 (4)	C33—C34—C35—Cl1	176.4 (4)
N2—C10—C11—C12	11.2 (3)	N2—C10—C36—O1	16.0 (4)
C36—C10—C11—C12	127.4 (3)	C9—C10—C36—O1	136.2 (3)
C9—C10—C11—C12	−109.4 (3)	C11—C10—C36—O1	−98.0 (3)
C30—C11—C12—N3	131.4 (2)	N2—C10—C36—O2	−165.9 (2)
C10—C11—C12—N3	−101.0 (2)	C9—C10—C36—O2	−45.6 (3)
C30—C11—C12—C13	−113.4 (3)	C11—C10—C36—O2	80.1 (3)
C10—C11—C12—C13	14.3 (3)	C7—C8—N1—C1	179.7 (4)
N3—C12—C13—N2	84.1 (3)	C3—C8—N1—C1	0.1 (4)
C11—C12—C13—N2	−35.6 (3)	C2—C1—N1—C8	−0.4 (4)
N3—C12—C13—C14	−42.0 (3)	C14—C13—N2—C10	172.5 (2)
C11—C12—C13—C14	−161.7 (2)	C12—C13—N2—C10	44.1 (3)
N2—C13—C14—N4	72.2 (3)	C36—C10—N2—C13	−150.8 (2)
C12—C13—C14—N4	−166.9 (2)	C9—C10—N2—C13	88.8 (2)
N2—C13—C14—C15	172.8 (3)	C11—C10—N2—C13	−34.6 (3)
C12—C13—C14—C15	−66.3 (4)	C11—C12—N3—O4	−157.2 (3)
N4—C14—C15—C24	113.5 (3)	C13—C12—N3—O4	89.3 (3)
C13—C14—C15—C24	−3.7 (5)	C11—C12—N3—O3	25.3 (4)
N4—C14—C15—C16	−3.9 (3)	C13—C12—N3—O3	−88.2 (3)
C13—C14—C15—C16	−121.1 (3)	O5—C16—N4—C17	16.9 (7)
C24—C15—C16—O5	63.8 (6)	C15—C16—N4—C17	−163.0 (3)
C14—C15—C16—O5	−175.6 (5)	O5—C16—N4—C14	175.3 (5)
C24—C15—C16—N4	−116.3 (3)	C15—C16—N4—C14	−4.5 (3)
C14—C15—C16—N4	4.2 (3)	C22—C17—N4—C16	−36.5 (5)
C22—C17—C18—C19	2.4 (5)	C18—C17—N4—C16	140.0 (4)
N4—C17—C18—C19	−174.1 (3)	C22—C17—N4—C14	173.4 (3)
C17—C18—C19—C20	0.8 (6)	C18—C17—N4—C14	−10.1 (6)
C18—C19—C20—O6	176.6 (4)	C13—C14—N4—C16	123.8 (3)
C18—C19—C20—C21	−3.5 (6)	C15—C14—N4—C16	4.4 (3)
C19—C20—C21—C22	3.2 (6)	C13—C14—N4—C17	−79.3 (4)
O6—C20—C21—C22	−176.9 (4)	C15—C14—N4—C17	161.3 (4)
C20—C21—C22—C17	−0.1 (7)	O1—C36—O2—C37	−1.3 (5)
C18—C17—C22—C21	−2.7 (6)	C10—C36—O2—C37	−179.4 (3)
N4—C17—C22—C21	173.9 (4)	C19—C20—O6—C23	−4.1 (6)
C16—C15—C24—C25	29.9 (5)	C21—C20—O6—C23	176.0 (4)
C14—C15—C24—C25	−69.4 (5)		

### Hydrogen-bond geometry (Å, °)

**Table:**

*D*—H···*A*	*D*—H	H···*A*	*D*···*A*	*D*—H···*A*
C11—H11···Cl1	0.98	2.57	3.095 (4)	114
C11—H11···O3	0.98	2.37	2.786 (4)	105
C22—H22···O5	0.93	2.59	3.080 (6)	113
C14—H14···O4^i^	0.98	2.53	3.443 (5)	154
C34—H34···O4^ii^	0.93	2.59	3.414 (6)	148
N1—H1A···O6^iii^	0.86	2.14	2.982 (5)	167

Symmetry codes: (i) −*x*, −*y*, −*z*; (ii) −*x*, −*y*+1, −*z*; (iii) −*x*+1, −*y*, −*z*+1.
